# Prediction of local COVID-19 spread in Heidelberg

**DOI:** 10.12688/f1000research.23034.1

**Published:** 2020-04-02

**Authors:** Lisa Koeppel, Claudius Gottschalk, Andreas Welker, Britta Knorr, Claudia M. Denkinger

**Affiliations:** 1 Division of Tropical Medicine, Heidelberg University, Heidelberg, Germany; 2 Gesundheitsamt des Rhein-Neckar-Kreises, Heidelberg, Germany

**Keywords:** COVID-19, compartmental model, mortality, hospitalization

## Abstract

Since the first identified case of COVID-19 in Wuhan, China, the disease has developed into a pandemic, imposing a major challenge for health authorities and hospitals worldwide. Mathematical transmission models can help hospitals to anticipate and prepare for an upcoming wave of patients by forecasting the time and severity of infections. Taking the city of Heidelberg as an example, we predict the ongoing spread of the disease for the next months including hospital and ventilator capacity and consider the possible impact of currently imposed countermeasures.

## Introduction

Since the first appearance of the novel coronavirus disease (COVID-19) in Wuhan, China, in December 2019, the disease has spread worldwide to be classified as a pandemic by the WHO on 11 March 2020.

In Germany the first confirmed COVID-19 case was recorded on 28 January 2020. Contact tracing and isolating of affected individuals interrupted the chains of transmission. However, with tourists returning from holidays in high-risk regions of northern Italy combined with a carnival celebration near Cologne, the virus spread to 13 out of 16 federal states within only a month ^1^. The exponential increase in new confirmed cases in Germany has reached 16,290 positively tested cases by 20 March 2020, with many more cases presumed undetected due to a possible transmission from minimally symptomatic or asymptomatic persons carrying the virus ^2, 3^.

The spread of COVID-19 in other countries, such as China and Italy, indicate the major challenges German hospitals, especially intensive care facilities, will have to face in the weeks to come. On 17 March 2020, the Robert Koch Institute declared the population risk as “high”. For the elderly and individuals with pre-existing health conditions, there is an increased probability of severe disease requiring hospitalization and critical care treatment, as well as higher mortality rates ^4^.

The spread of the disease varies locally and depends on several factors, such as the temporal progression of the infection in the specified region, the size and the age distribution of the population, the available capacities and the implemented countermeasures. Thus, to prepare hospitals and assist health authorities in the most efficient way, the resulting challenge and burden on the health care system should be modelled locally. As an exemplary case, in this manuscript we are concerned with the spread of the COVID-19 epidemic for the city of Heidelberg in Germany.

The first COVID-19 positive case in Heidelberg was confirmed on 28 February 2020, with eight more cases sporadically appearing in the following two weeks ^5^. The vast majority were home-comers from vacations in a high-risk region in Italy and their immediate contacts. All positive tested individuals were isolated and several hundred contacts were traced and quarantined to prevent further spread. Only thereafter, the total numbers of positively tested COVID-19 cases rose to 20 on March 15, and 34 on March 16.

The capabilities to cope with the increasing burden on the health system on a local scale depend on the preparedness of available local resources in the hospitals and the implemented countermeasures (isolation, quarantine, social distancing) to slow down the disease spread. In such an extraordinary situation, it is crucial for hospitals to rely on future predictions about the upcoming need of beds as well as ventilators. Predictions from mathematical modelling can assist authorities in their decision making on suitable countermeasures to slow down the viral transmission and prepare the health systems.

## Methods

### The transmission model and the parameters

We model the transmission dynamics of the COVID-19 outbreak by an individual level susceptible (S) – exposed (E) – infected (I) – removed (R; SEIR) compartmental model. The transitions between the compartments are described through the following set of differential equations

$$\frac{dS}{dt}=-R_{0}\gamma SI$$

$$\frac{dE}{dt}=R_{0}\gamma SI-\kappa E$$

$$\frac{dI}{dt}=\kappa E-\gamma I$$

$$\frac{dR}{dt}=\gamma I$$

Where *γ* is the inverse of the infectious period and *κ* is the inverse of the incubation period. *Extended data*, Table 1 ^6^ lists all parameter choices for our model. For the main part of this manuscript we assume an incubation period of 5 days ^7^ and an infectious period each of 2.9 days ^8^. The infectiousness of the disease is given by *R*_0_ and is defined as the mean number of people that get infected by a single infected individual over the whole infectious period. Due to the limited data about the COVID-19 pandemic, there is much uncertainty in the estimation of *R*_0_. As baseline we follow the value of 2.2 obtained from the COVID-19 outbreak on the Diamond Princess ship ^9^. For a sensitivity analysis, we also consider a lower *R*_0_ of 1.5 with respect to the estimation of Du *et al.* (2020) ^10^ and a more severe outbreak progression with 3 in accordance to the estimation of *R*_0_ ranging between 2.2 and 3.6 for the SARS epidemic in 2003 ^11^.

According to the office for statistics and urban development Heidelberg ^12^, for our model we assume a closed population of 150,000, without individual entering or leaving the system. We consider this as a realistic scenario because we do not expect major demographic changes to occur within the six months of the projections.

The true number of initial infected individuals remains unknown due to the asymptomatic progression in some cases. Furthermore, due to limited testing capacities, only high-risk individuals (i.e. people who had prior close contact to infected individuals or people returning from a high risk region according to the WHO definition) were tested, resulting in missing positive cases in the population that resulted from possible community transmission. To capture this uncertainty, we assume that the outbreak starts on 12 March 2020 with 10 initial infectious individuals who have not been isolated and are able to transmit the disease.

## Results

**Figure 1.**
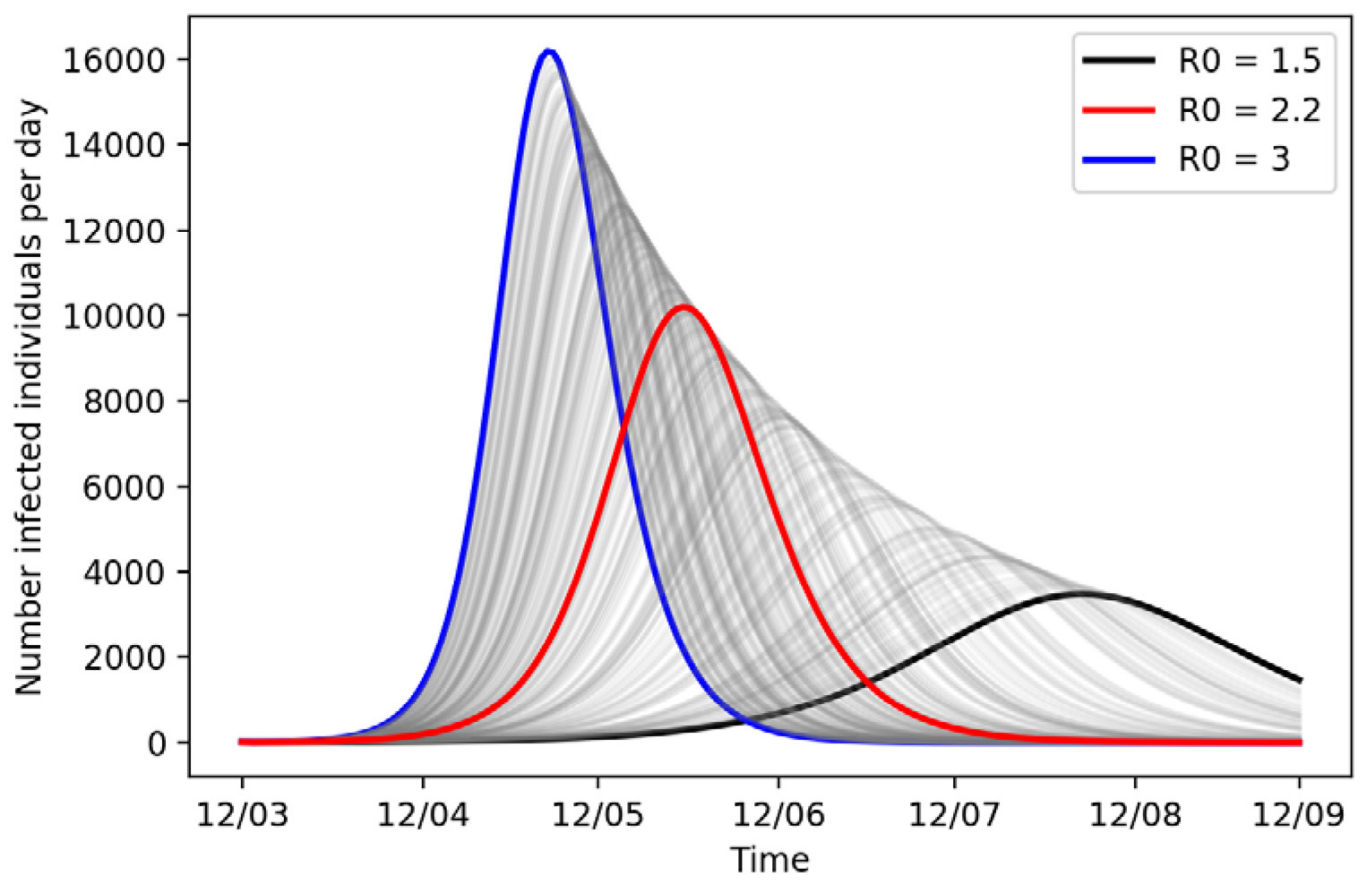
Number of infected individuals per day for values of *R*_0_ between 1.5 and 3 for sensitivity analysis. In all simulations, the number of initial infections was 10 and the mean incubation and infectious period is 5 days.

For now, we assume that no countermeasures have been set in place and predict the future dynamics of the outbreak for the upcoming period of 6 months. Figure 1 displays the number of infected individuals per day for varying values of *R*_0_. In the baseline scenario ( *R*_0_ = 2.2) the peak of the infection will be 10196 individuals infected on 26 May and a total outbreak size of 126,574 (84%) of the population infected after six months. This aligns with estimates for the proportion of British people get infected if no countermeasures are put in place ^13^.

In the mild outbreak scenario, there will be 77952 (52%) total infections by 12 September 2020, whereas in the severe scenario we will have a rapid growth and a shorter doubling of infections time with 141,084 (94%) infections in total.

For sensitivity analysis, we also investigated, with respect to expert opinion, shortening and prolonging the incubation period [3.5–5.5 days] as well as the infectious period [1.5–4.5 days] (see *Extended data*, Figure A1 ^6^). The model showed more robustness to this kind of variation on the overall outbreak curve with a shift in max 42 days for the peak of the outbreak curve and difference of 8856 infections during peak time (compared to 90 days and 12,635 when varying *R*_0_).

When varying the number of first infected individuals, the dynamics of the outbreak stay the same. The more infected individuals we start the simulation with, the further we assume the outbreak has progressed already (see *Extended data*, Figure A2 ^6^).

### Countermeasures for reducing transmission

From these scenarios, it is obvious that lowering *R*_0_ leads to a lowering of the final size of the epidemic, prolonging the peaking and minimizing the peak. The latter is the most important, as it avoids an outbreak trajectory, in which a large number of people get sick at the same time with overwhelming effects for the health system.

In the hope to lower the amount of secondary cases resulting from one primary case, on 22 March 2020, the German government announced the implementation of extraordinary measures to reduce social contact and thus transmission for at least two weeks. These include regulations on social distancing such as the closure of schools, kindergartens, shops, restaurants, imposing a visiting ban for hospitals and elderly homes, as well as preventing transmission amplification events and social interactions in public with more than two persons. Such countermeasures can be considered in the model by diminishing the transmission intensity *R*_0_.

**Figure 2.**
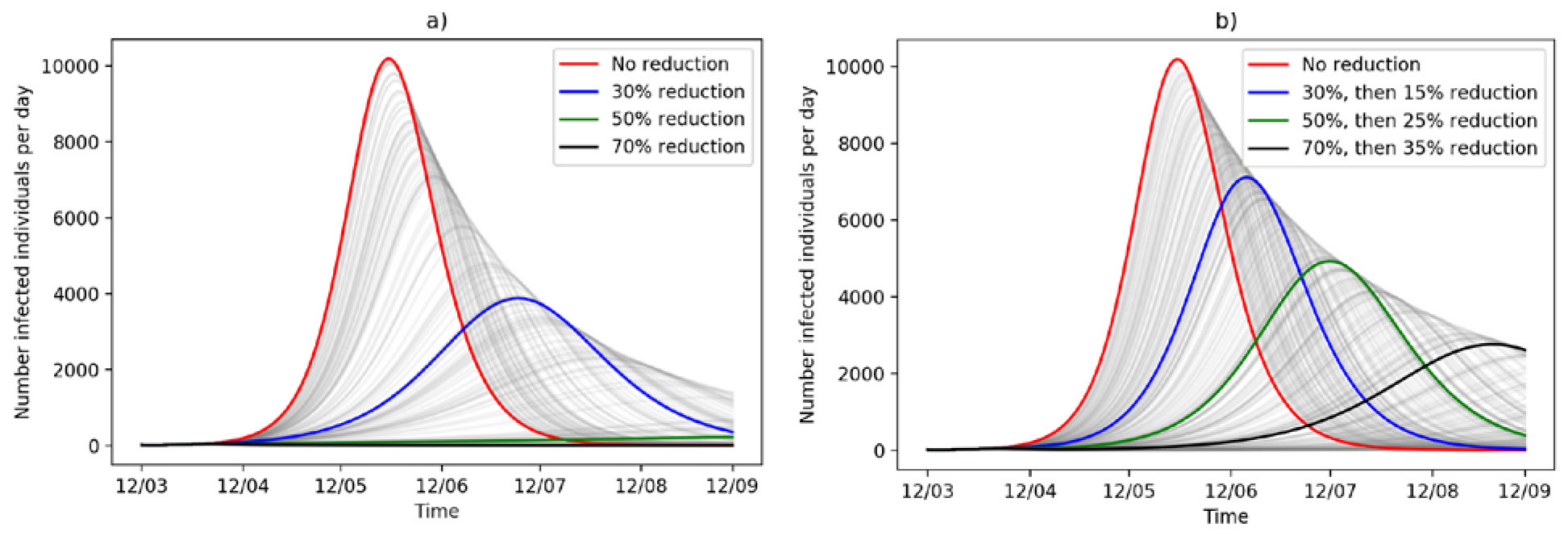
Possible outbreak progressions with reduction in social contact, thus disease transmission, after Monday, 23 March 2020. The baseline transmission rate is *R*_0_ = 2.2. (a) Permanent reduction of disease transmission over the next six months. (b) After three weeks of reduced transmission, transmission increases again by 50% of the reduction.

Figure 2 displays the progression of the outbreak in Heidelberg with respect to different reductions in social contact and thus disease transmission from Monday, March 23 onwards. It illustrates, that restricting social contact will have a major impact on the number of infected individuals per day, on the overall outbreak size and flatten the curve. In fact, a permanent transmission reduction by 30% will yield a total of 89,022 infected individuals by mid-September and 3,877 during the peak time ( Figure 2a). Decreasing social contact by 50% will massively flatten the curve, with a total of 6,927 and 223 infected individuals at peak time. This will not stop the outbreak, but it will slow it down to a major extent. Considering the transmission on a scale of one year in time, with the permanent reduction of 50% of transmission, we will have 15% of the population of Heidelberg infected.

However, such a permanent lock-down of the population will not be feasible due to economical and psychological health reasons. Thus, in Figure 2b, we assume that after three weeks social life will be partially resumed and transmission rates will increase again by 50%. In this case, a transmission reduction by 70% at first with a following increase in contact rates (transmission reduction lowered to 35%) will lead to a total of 48,468 people infected by September. Compared to the worst-case scenario (permanent *R*_0_ = 2.2) this is a decrease of 62% (78,106) in the total number of people infected.

If after three weeks contact rates will revert to their original state, the outbreak curve will only be shifted by weeks with no effect on the height nor on the outbreak size (see Extended data, Figure A3 ^6^).

### Deaths, hospitalizations and ventilator capacity

**Figure 3.**
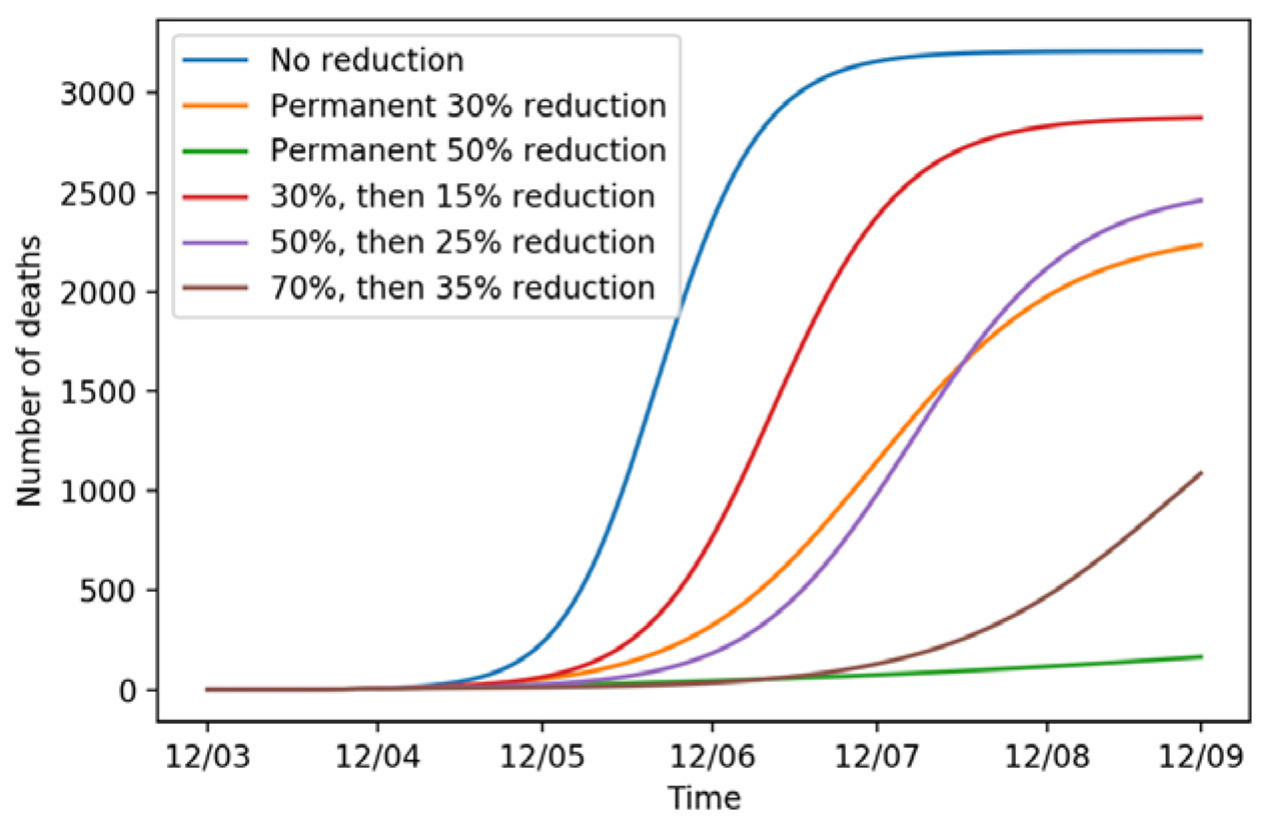
Expected number of cumulative deaths from COVID-19 in Heidelberg in the next six months.

As the severity of the disease and thus the number of expected deaths from the COVID-19 outbreak depends on the age of the individual, we obtained the specific age distribution for the city of Heidelberg: <55 (72.4%), 55–65 (11.06%), 65–75 (7.77%), and >75 years of age (8.77%) ^12^. Adapting from Dowd *et al.* (2020) ^14^, the mortality probabilities for each age category are 0.1% (<55), 1.5% (55–65), 7% (65–75), and 20% (>75 years of age). By means of these percentages we calculated the cumulative number of deaths in Heidelberg as proportions of the people in the “Removed (R)” compartment over time ( Figure 3) for different scenarios of transmission reduction. We model the delay between end of infectious period and time of death by 6.2 days and follow Sanche *et al.* (2020) ^15^, who assume that the duration from hospital admittance to death is 11.2 days.

As preparation for the health system to plan the bed and ventilator capacity that needs to be enabled, it is important to predict the number of patients who require hospitalization and ventilation.

**Figure 4.**
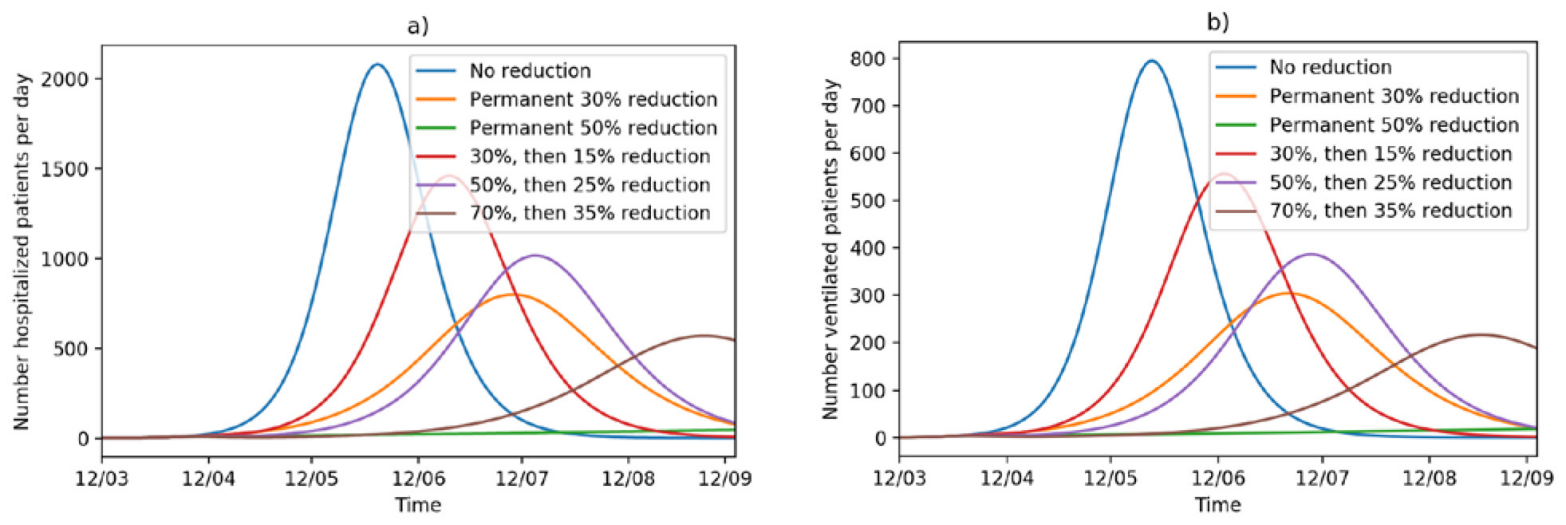
Number of hospitalized patients and number of ventilated patients per day.

It is difficult to give estimates for the proportion of hospitalization from all infected individuals, because there is an inherent bias in the existing data ^16^. In the beginning of an epidemic, people are hospitalized for isolation purposes but not because of their severe symptoms. Besides, due to asymptomatic or minimally symptomatic infections that do not present for testing, the true number of infections remains unknown and thus estimates of hospitalization might be overly pessimistic. For this simulation study, we nevertheless assume, that 5% of all infected individuals need hospitalization. Because the duration of hospitalization varies in the literature, we take the smaller and more conservative estimate of 11.5 days ^15, 17, 18^ ( Figure 4a). As the curves are proportions of the number of infected individuals, changing the proportion of hospitalizations will only change the scale of the plot. A sensitivity analysis reducing the duration of hospitalization to seven days is shown in Figure A3 in the appendix.

For calculating the number of ventilators needed, we assume that 90% of all people who died from the disease were ventilated before. The remaining 10% possess a patient do not resuscitate/do not intubate (DNR/DNI) status. We further assume that 60% of ventilated die. This leads to 1.5 (0.9/0.6) people ventilated per death count. According to Chen *et al.* (2020) ^19^, the average duration of ventilation is 3-20 days. Supposing a conservative estimate of 7 days, we can model the capacity in the Intensive Care Unit given the calculated death numbers.

According to the Science Media Center Germany, routine capacity for ventilation of the University Hospital Heidelberg is 112 spaces ^20^. Even if we unrealistically assume that all spaces can be used for COVID-19 patients, the capacity will not be enough within the following six months, unless we have a permanent reduction in the disease transmission by 50% ( Figure 4b).

## Discussion

This manuscript modelled the future of COVID-19 spread within the city of Heidelberg and showed the various possible scenarios over the coming weeks and months. Considering the current exponential growth of cases in Germany ^1^, we have to assume a current *R*_0_ between 2.2. and 3.

Our model has several limitations. Our model does not account for the percentage of people with previous conditions who are at increased risk. We also did not include any movement data in our model and did not account for tourists visiting Heidelberg, which we assume to be a realistic scenario given that governmental restrictions will keep movement to a minimum in the following months. We also did not account for deaths due to selective treatment of COVID-19 patients over others with different health conditions when bed capacity is exceeded.

Although the number of infected individuals and therefore the number of deaths can be reduced by prolonging the outbreak, severe impacts on the economy as well as effects on psychological and social life can be expected if restrictions on social behaviour and interactions will be imposed for a longer time. In an optimistic scenario with a permanent reduction in transmission, in twelve months 50% of the population in Heidelberg will be infected. This clearly states the urgency for developing and distributing vaccines among the population.

The predictions of our model are estimations and include inherent uncertainty because the model parameters are derived from only limited clinical and epidemiological data of the COVID-19 outbreak dynamics and estimates thereof. Moreover, the future behaviour of the population, their adherence to movement and social contact restriction and the timely development of a suitable vaccine determine the ongoing of the spread. To better understand, where we are on the epidemic curve, requires more testing. The currently implemented restrictive testing strategy focuses on people returning from high-risk regions and their immediate contacts and does not capture ongoing community transmission. Community transmission is likely going to be more relevant for transmission at this stage of the epidemic as it is more difficult capture and more dispersed. Currently available testing capacity would not allow testing of all persons with respiratory symptoms in the city of Heidelberg, but at a minimum sentinel testing of a representative subset of the population should be considered. Ultimately a seroprevalence survey will be necessary to completely understand the true number of cases in the population.

We anticipate the modelling outcomes can be useful for health authorities in their decision-making process to plan resources and bed capacities ahead of time and implement suitable preparations to meet the upcoming medical need.

## Data availability

### Underlying data

All data underlying the results are available as part of the article and no additional source data are required.

### Extended data

Figshare: Prediction of local COVID-19 spread in Heidelberg - Supplementary Material. https://doi.org/10.6084/m9.figshare.12038664 ^6^.

This project contains the following extended data:

- Figure A1: 500 Simulations with fixed R0=2.2 and changing values for κ~U [0.1818,0.2857] and γ~U [0.2222,0.6666].
- Figure A2: Outbreak simulations with varying number of first infected individuals on 12 March 2020.
- Figure A3: Outbreak simulations with different reductions in transmission from Monday, 23 March 2020.
- Table 1: Choices of model parameters.

Extended are available under the terms of the Creative Commons Attribution 4.0 International license (CC-BY 4.0).
