# Supplementary material for: Prediction of local COVID-19 spread in Heidelberg
Source: F1000Res. 2020 Apr 2;9:232. [Version 1] doi: 10.12688/f1000research.23034.1 (PMC7445560; doi:10.12688/f1000research.23034.1)
Supplement: Peer review history

# Peer review: Reviewer response for version 1

**DOI:** 10.5256/f1000research.25429.r69089

**Published:** 2020-08-24

**Authors:** Farhad Hatami

**Affiliations:** 1 Centre for Health Informatics, Computation and Statistics, Lancaster Medical School, Lancaster University, Lancaster, UK

In Koppel *et al.*, the authors propose a SEIR (Susceptible, Exposed, Infected, Removed or Recovered) differential equations model estimating the spread of COVID-19 in the city of Heidelberg.

 The proposed modelling approach is performed on a closed population with no new entry/leave. As declared by the authors, a more realistic approach could extend the current model with taking into account the scenario where people with different underlying health conditions/immune systems would have an unexpectedly higher rate of susceptibility/infection, or a second scenario where a "recovered" case does not necessarily mean "not susceptible" anymore. However, for the sake of this paper, the deployed model benefits from high robustness which makes it a reliable prediction algorithm.

 A sensitivity analysis of the model has been performed via applying on a range of assumptions for countermeasures and infection rates. As a result, changing the number of initially infected individuals at the beginning of the pandemic seems to only shift the distribution linearly with no effect on the peak. Considering the limitations in the intensive care unit, a set of policies have been introduced to help the authorities in Germany. 

 While using a well-known robust algorithm, and bearing in mind that this is not supposed to be a methodological research, the paper still lacks some validation tests. A validation test on the existing benchmarking experiment can be carried out in several ways. At the time of this review, there might be access to the real data of COVID-19 spread in Heidelberg in order to compare with the estimated results. Alternatively, the same approach (of course with a different rate of daily infection) could be applied on a data of SARS to test the performance of the model.

Is the work clearly and accurately presented and does it cite the current literature?

Yes

If applicable, is the statistical analysis and its interpretation appropriate?

Yes

Are all the source data underlying the results available to ensure full reproducibility?

Partly

Is the study design appropriate and is the work technically sound?

Yes

Are the conclusions drawn adequately supported by the results?

Yes

Are sufficient details of methods and analysis provided to allow replication by others?

Yes

Reviewer Expertise:

Biostatistics, Machine Learning

I confirm that I have read this submission and believe that I have an appropriate level of expertise to confirm that it is of an acceptable scientific standard.

# Peer review: Reviewer response for version 1

**DOI:** 10.5256/f1000research.25429.r68119

**Published:** 2020-08-10

**Authors:** Arindam Basu

**Affiliations:** 1 School of Health Sciences, University of Canterbury, Canterbury, UK

The authors used a compartmental model that they referred to as a SEIR model where they divided the population into four compartments: Susceptible (S), Exposed (E), Infected (I), and Removed (R; Removed == "Recovered" or "Died", hence cannot be reinfected, although this assumption could be modified but for the sake of argument, we will accept that those who recovered could not be reinfected and hence removed). 

 Now the model could be specified differently as their scheme of the equations was not clear to me. The susceptibles pool depleted at a constant rate of exposure (typically mixing rates and rate of contact with another infected person), but they seem to have substituted and mixed up with an SIR model (susceptible, infected, and Recovered/Removed model). 

 S -> E -> I -> R should be the correct order of their model specification, so one way to formulate would be

 dS/dt = - Rate of Exposed

 dE/dt = Rate of Exposed - Rate of Infected

 dI/dt = Rate of Infected - Rate of removal

 dR/dt = Rate of accumulation of removed persons from the pool

 But from reading their formulae it does not appear that they went with these equations, nor did they explain the basis or provide a reproducible code.

 Hence, although the epidemic curves look plausible, it is difficult to interpret how they were specified or their true significance. The authors may want to clarify this aspect of the methods section.

Is the work clearly and accurately presented and does it cite the current literature?

Partly

If applicable, is the statistical analysis and its interpretation appropriate?

Partly

Are all the source data underlying the results available to ensure full reproducibility?

No

Is the study design appropriate and is the work technically sound?

Partly

Are the conclusions drawn adequately supported by the results?

No

Are sufficient details of methods and analysis provided to allow replication by others?

No

Reviewer Expertise:

Epidemiology

I confirm that I have read this submission and believe that I have an appropriate level of expertise to confirm that it is of an acceptable scientific standard, however I have significant reservations, as outlined above.

# Peer review: Reviewer response for version 1

**DOI:** 10.5256/f1000research.25429.r68118

**Published:** 2020-08-10

**Authors:** Mohammad Reza Mahmoud

**Affiliations:** 1 Department of Statistics, Faculty of Science, Fasa University, Fasa, Iran

In this work, the authors predict the ongoing spread of the disease including hospital and ventilator capacity for the next months in the city of Heidelberg and consider the possible impact of currently imposed countermeasures.

 This is an interesting contribution to the existing literature, but the paper suffers from several shortcomings listed in the following comments:

- The paper should be checked by a native.
- A Conclusion section should be added.

- The novelty and contribution should be clearly bolded.

- The introduction should be updated by recent researches. The authors should consider some works about statistical methods (including time series models or regression models) that can be applied to model and predict medical datasets.
- Comparative Analysis should be given in more details.
- It’s better to suggest some subjects for future works.

Is the work clearly and accurately presented and does it cite the current literature?

Yes

If applicable, is the statistical analysis and its interpretation appropriate?

Yes

Are all the source data underlying the results available to ensure full reproducibility?

Yes

Is the study design appropriate and is the work technically sound?

Yes

Are the conclusions drawn adequately supported by the results?

Partly

Are sufficient details of methods and analysis provided to allow replication by others?

Yes

Reviewer Expertise:

Data Analysis, Statistics, Data Modelling

I confirm that I have read this submission and believe that I have an appropriate level of expertise to confirm that it is of an acceptable scientific standard, however I have significant reservations, as outlined above.
